# Prevalence and Determinants of Dementia Among Older Adults Attending Outpatient Clinics in a Tertiary Care Hospital in Thailand: A Secondary Data Analysis

**DOI:** 10.7759/cureus.91454

**Published:** 2025-09-02

**Authors:** Kasidid Lawongsa, Supanut Kumjan

**Affiliations:** 1 Department of Family Medicine, Phramongkutklao Hospital, Bangkok, THA; 2 Division of Hematology, Department of Medicine, King Chulalongkorn Memorial Hospital, Bangkok, THA

**Keywords:** dementia, outpatient clinics, prevalence, risk factors, secondary data, tertiary hospital, thailand

## Abstract

Background: Dementia is an increasing public health concern, particularly in aging societies. While most prevalence estimates in Thailand are derived from community-based studies, less is known about the burden and risk factors of dementia in tertiary care outpatients, who often present with multimorbidity.

Methods: We conducted a retrospective cross-sectional study using secondary data from patients aged ≥60 years attending internal medicine, neurology, and geriatric outpatient clinics of Phramongkutklao Hospital, a tertiary hospital in Bangkok, Thailand, between January 1 and December 31, 2024. Dementia was identified using a hierarchical algorithm incorporating International Classification of Diseases, Tenth Revision (ICD-10) diagnostic codes, prescriptions for anti-dementia medications, and cognitive test results, with sensitivity analyses using stricter definitions. Independent variables included demographic characteristics, vascular and non-vascular comorbidities, medication use, and laboratory results. Prevalence was calculated with 95% confidence intervals (CI). Poisson regression with robust variance estimation was used to calculate crude and adjusted prevalence ratios (aPR) for associated factors.

Results: Of 4,100 older adults, 512 had dementia (12.5%). Alzheimer’s disease was the most frequent subtype, accounting for 278 cases (54.3% of dementia; 6.8% of the total sample). In univariable analysis, dementia was associated with age ≥80 years, female sex, low education, hypertension, diabetes mellitus, stroke/transient ischemic attack (TIA) history, depression, and an Anticholinergic Cognitive Burden (ACB) score ≥3. After adjustment, age ≥80 years remained the strongest determinant (aPR 2.41, 95% CI 1.92-3.01). Female sex (aPR 1.28, 95% CI 1.07-1.53), education ≤6 years (aPR 1.64, 95% CI 1.33-2.01), hypertension (aPR 1.21, 95% CI 1.02-1.43), diabetes mellitus (aPR 1.27, 95% CI 1.07-1.52), stroke/TIA (aPR 1.89, 95% CI 1.52-2.35), depression (aPR 1.48, 95% CI 1.16-1.90), and ACB score ≥3 (aPR 1.36, 95% CI 1.09-1.70) were all independently associated with dementia.

Conclusion: Dementia was common among tertiary care outpatients, with prevalence higher than community-based estimates. Advanced age, female sex, low education, vascular comorbidities, depression, and anticholinergic medication burden were significant correlates. These findings highlight the need for routine screening, aggressive vascular risk factor management, and medication review in tertiary care settings to mitigate the burden of dementia.

## Introduction

Dementia is a major global public health concern characterized by progressive cognitive decline, impaired daily functioning, and increased dependency. In 2019, the number of people living with dementia worldwide was estimated at 55 million, and this figure is projected to rise to 139 million by 2050, largely driven by population aging in low- and middle-income countries (LMICs) [1,2]. The global economic burden is substantial, with annual costs estimated at US$1.3 trillion [3].

In Asia, the prevalence of dementia has been increasing rapidly over the past two decades. A meta-analysis of studies in East and Southeast Asia reported pooled prevalence rates ranging between 6% and 10% among adults aged 60 years and older [3]. In Thailand, community-based studies have demonstrated prevalence ranging from 2.8% to 8.1%, with variation across regions [4,5]. However, most existing data are derived from community surveys, while clinic-based data in tertiary care hospitals remain scarce. Given that hospital populations often include individuals with multiple comorbidities, such as hypertension, diabetes, and stroke, the prevalence and risk profile of dementia in clinical settings may differ substantially from community-based estimates [6-8].

Several demographic and clinical factors are consistently associated with dementia. Non-modifiable risk factors include advanced age, female sex, and sex [6,7]. Vascular risk factors, such as hypertension, diabetes mellitus, dyslipidemia, atrial fibrillation, and prior stroke, are strongly linked to dementia incidence [7,8]. Other contributors include depression, social isolation, smoking, physical inactivity, and hearing loss, all of which are common in older populations [9,10]. While these associations are well established in community studies, their impact on tertiary care outpatients, where multimorbidity and polypharmacy are more prevalent, has been less well characterized.

Tertiary care hospitals provide a unique context for studying dementia, as they encompass diverse outpatient clinics, including internal medicine, neurology, and geriatrics, that attract patients with varying comorbidity profiles and cognitive concerns. Secondary data from hospital medical records and electronic health systems can therefore offer valuable insights into both the prevalence of dementia and its associated risk factors in real-world clinical practice. Such evidence is critical for developing effective screening strategies, optimizing resource allocation, and informing tailored interventions in high-risk groups within healthcare systems.

Therefore, this study aimed to determine the prevalence of dementia among older adults attending outpatient clinics of a tertiary care hospital and to identify associated risk factors using secondary clinical data.

## Materials and methods

This study was designed as a retrospective cross-sectional analysis using secondary data obtained from outpatient clinics of Phramongkutklao Hospital, a tertiary care hospital in Bangkok, Thailand. The hospital provides comprehensive services, including internal medicine, neurology, and geriatric clinics, which together serve a large and diverse population of older adults.

The study population included all patients aged 60 years and older who attended at least one outpatient visit in the selected clinics between January 1 and December 31, 2024. For individuals with multiple visits during the study period, the first visit was defined as the index visit. Patients were eligible if they were aged 60 years or above at the time of the index visit and attended one of the selected outpatient clinics. Patients were excluded if demographic information such as age or sex was missing, if documentation indicated terminal illness or palliative care at the index visit, or if the record contained implausible values, such as age greater than 110 years or negative time intervals.

Dementia was defined using a hierarchical algorithm based on electronic medical records. Patients were considered to have dementia if they met at least one of the following criteria: (1) the presence of at least one International Classification of Diseases, Tenth Revision (ICD-10) dementia code recorded as a primary diagnosis (G30*, F01*, F03*, G31.83, or F02*), or at least two mentions of such codes in any diagnosis field within 12 months [11]; (2) prescription of cholinesterase inhibitors (donepezil, rivastigmine, or galantamine) or memantine on at least two occasions separated by 30 days or more (To reduce the risk of outcome misclassification, we applied a composite definition for dementia. In the case of anti-dementia drugs, a diagnosis requires at least two prescriptions of cholinesterase inhibitors or memantine issued more than 30 days apart. This approach was intended to capture ongoing treatment rather than a single or trial prescription. Although the analyses were anchored at the index visit, medication records across the observation period were reviewed to establish case status; or (3) cognitive test results, where the Thai version of the Mini-Mental State Examination (TMSE) score [12] was less than 24 or the Montreal Cognitive Assessment (MoCA) score [13] was less than 26, documented by the treating physician. Patients meeting any of these criteria were classified as having dementia. Cognitive tests (TMSE or MoCA) were recorded only when clinically indicated, rather than systematically administered to all patients without a diagnostic code or prescription. Sensitivity analyses were also performed using more restrictive definitions, such as requiring ICD-10 codes in the primary diagnosis only or combining ICD-10 codes with prescription records.

Independent variables were obtained from hospital databases and included demographic, clinical, and lifestyle-related factors. Demographic variables were age, sex, marital status, and education level when available. The type of health insurance was also collected as a proxy measure for socioeconomic status, but because these data were incomplete for a number of participants, they were not included in the descriptive summary. Comorbidities were identified using ICD-10 codes recorded within the two years preceding the index visit and included hypertension, diabetes mellitus, stroke or transient ischemic attack, and depression. At the index visit, we reviewed each patient’s current outpatient prescriptions and calculated the Anticholinergic Cognitive Burden (ACB) score from the active medications recorded at that time, including antihypertensives, statins, antiplatelets, anticoagulants, antidiabetic drugs, benzodiazepines, and anticholinergic drugs. The latter was used to calculate an ACB score [14]. The TMSE, MoCA, and ACB scores are open-access tools. Each medication was assigned an ACB score according to its level of anticholinergic activity. A score of one was given to drugs with possible anticholinergic effects, usually identified through serum assays, receptor affinity, or in vitro studies. A score of two was assigned to medications with definite anticholinergic effects supported by clinical data, though their impact is considered less severe. A score of three was reserved for medications with well-established anticholinergic properties that are strongly associated with clinically relevant cognitive impairment. The total ACB score was then calculated by summing the scores of all medications a patient is taking, providing an estimate of their cumulative anticholinergic burden. Vital signs and laboratory results within six months of the index visit were collected, including body mass index (BMI), blood pressure, fasting plasma glucose, HbA1c, lipid profile, and estimated glomerular filtration rate (eGFR). Additional tests, such as thyroid-stimulating hormone (TSH), vitamin B12, and folate, were reviewed when available to help rule out reversible causes of cognitive impairment or dementia mimics. The primary outcome was dementia status, defined as either present or absent at the index visit according to the criteria described above.

Descriptive statistics were used to summarize baseline characteristics. Continuous variables were reported as means with standard deviations (SD) or medians with interquartile ranges (IQR), while categorical variables were expressed as frequencies and percentages. The prevalence of dementia was calculated as the proportion of individuals meeting the dementia criteria out of the total eligible population and was reported both overall and stratified by clinic type. Exact binomial 95% confidence intervals (CIs) were calculated, and age- and sex-standardized prevalence estimates were derived using direct standardization with the study cohort as the reference.

Associations between potential risk factors and dementia were assessed using univariable analyses with χ² tests for categorical variables and t-tests or Mann-Whitney U tests for continuous variables. Multivariable Poisson regression with robust variance estimation was performed to calculate adjusted prevalence ratios (aPRs) and 95% CIs, which provide more interpretable estimates than odds ratios (ORs) in cross-sectional data [15]. The multivariable model included variables that were significant in the univariable analysis, together with factors considered clinically important from earlier studies. This approach helped balance statistical evidence with established clinical relevance [6-8,16-18].

The study protocol was approved by the Institutional Review Board of the Royal Thai Army Medical Department (approval number: IRBTA0269/2024). Because the study used secondary anonymized data, informed consent was waived. All procedures were conducted in accordance with the principles outlined in the Declaration of Helsinki.

## Results

**Figure 1:**
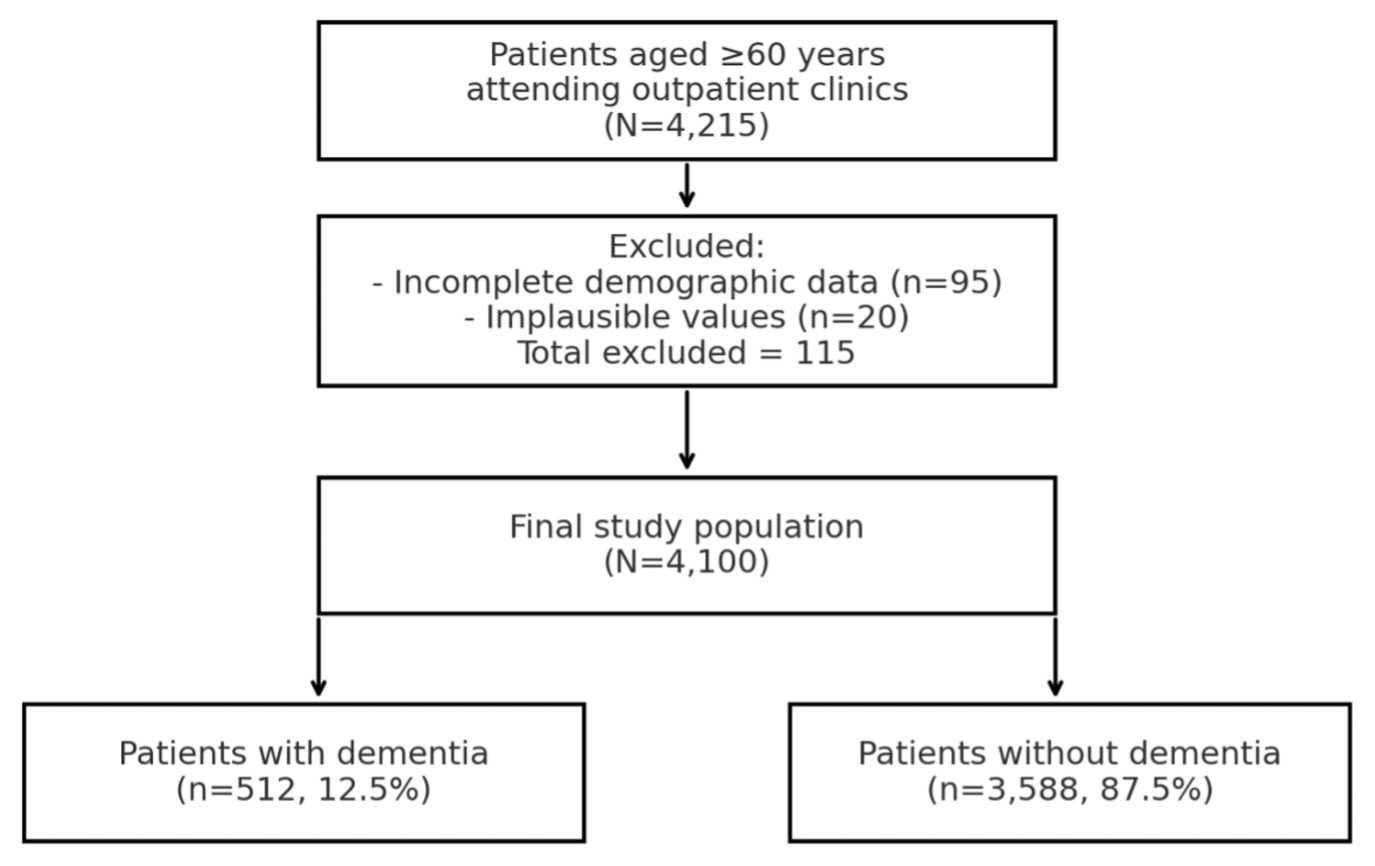
A flow diagram outlining the selection of study participants

A total of 4,215 patients aged 60 years and older attended at least one outpatient visit in the internal medicine, neurology, or geriatric clinics during the study period. After excluding 115 patients with incomplete demographic data or implausible values, 4,100 patients were included in the final analysis (Figure 1). The mean age of the study population was 71.8 years (SD 7.4), and 57.2% were female.

Of the 512 dementia cases in our cohort, Alzheimer’s disease was the most frequent subtype (278 cases, 54.3%), followed by vascular dementia (136 cases, 26.6%) and mixed dementia (54 cases, 10.5%). Less common forms included Lewy body dementia and frontotemporal dementia (18 cases, 3.5%), while unspecified dementia (ICD-10 F03) was recorded in 26 cases (5.1%) (Table 1).

**Table 1:** Prevalence of dementia subtypes among older adults (n = 4,100)

Dementia subtype	n (cases)	% of dementia (n=512)	% of total sample (n=4,100)
Alzheimer’s disease (F00)	278	54.30%	6.80%
Vascular dementia (F01)	136	26.60%	3.30%
Mixed dementia (F00+F01)	54	10.50%	1.30%
Other specified dementia (F02)	18	3.50%	0.40%
Unspecified dementia (F03)	26	5.10%	0.60%
Total	512	100%	12.50%

Baseline characteristics of participants by dementia status are shown in Table 2. On average, individuals with dementia were older than those without (76.3 vs. 71.1 years, p<0.001). Women made up 60.9% of the dementia group compared with 56.5% of the non-dementia group (p=0.04). Low education (≤6 years) was also more common among those with dementia (62.9% vs. 40.6%, p<0.001).

**Table 2:** Baseline characteristics of study participants by dementia status Values are presented as mean (standard deviation (SD)) for continuous variables and number (percentage) for categorical variables. TIA: transient ischemic attack; ACB: Anticholinergic Cognitive Burden.

Characteristic	Dementia (n=512)	No dementia (n=3,588)	Total (n=4,100)	p-value
Age, mean (SD), years	74.6 (6.8)	71.4 (7.5)	71.8 (7.4)	<0.001
Female sex, n (%)	312 (60.9)	2,028 (56.5)	2,340 (57.2)	0.04
Education ≤6 years, n (%)	322 (62.9)	1,458 (40.6)	1,780 (43.4)	<0.001
Marital status				<0.001
Married, n (%)	302 (59.0)	2,501 (69.7)	2,803 (68.5)	
Widowed, n (%)	170 (33.2)	790 (22.0)	960 (23.4)	
Single/divorced, n (%)	40 (7.8)	297 (8.3)	337 (8.1)	
Hypertension, n (%)	338 (66.0)	1,923 (53.6)	2,261 (55.3)	<0.001
Diabetes mellitus, n (%)	170 (33.2)	897 (25.0)	1,067 (26.1)	<0.001
Stroke/TIA history, n (%)	112 (21.9)	356 (9.9)	468 (11.4)	<0.001
Depression, n (%)	60 (11.7)	259 (7.2)	319 (7.8)	<0.001
BMI, mean (SD), kg/m²	23.2 (3.5)	24.3 (3.7)	24.1 (3.7)	<0.001
ACB score ≥3, n (%)	112 (21.9)	392 (10.9)	504 (12.3)	<0.001

Comorbidities differed significantly between groups. Hypertension was present in 66.0% of the dementia group versus 53.6% of the non-dementia group (p<0.001), and diabetes mellitus was more frequent in those with dementia (33.2% vs. 25.0%, p<0.001). A history of stroke or transient ischemic attack (TIA) was nearly twice as common in the dementia group (21.9% vs. 9.9%, p<0.001). Depression was also more often reported among participants with dementia (11.7% vs. 7.2%, p<0.001).

In addition, participants with dementia had a lower mean BMI (23.2 vs. 24.3 kg/m², p<0.001) and were more likely to have an ACB score ≥3 (21.9% vs. 10.9%, p<0.001).

As shown in Table 3, the univariable analysis demonstrated significant associations between dementia and several factors. Older age (≥80 years) was strongly related to higher prevalence (crude PR 2.98, 95% CI 2.45-3.63, p<0.001). Female sex was also associated with increased dementia prevalence (crude PR 1.24, 95% CI 1.05-1.47, p=0.01). Having six years of education or less was linked with nearly a twofold higher prevalence compared with higher education levels (crude PR 1.89, 95% CI 1.56-2.29, p<0.001). Hypertension (crude PR 1.35, 95% CI 1.14-1.59, p<0.001) and diabetes mellitus (crude PR 1.41, 95% CI 1.19-1.67, p<0.001) were both significant. A history of stroke or transient ischemic attack (TIA) showed one of the strongest crude associations (PR 2.41, 95% CI 1.99-2.92, p<0.001). Depression was also significant (crude PR 2.12, 95% CI 1.71-2.62, p<0.001). In addition, an ACB score ≥3 was associated with almost double the prevalence of dementia (crude PR 1.82, 95% CI 1.45-2.29, p<0.001).

After adjusting for potential confounders, these associations remained statistically significant, though some were attenuated (Table 3). Age ≥80 years continued to show the strongest effect (adjusted PR 2.41, 95% CI 1.92-3.01, p<0.001). Female sex (aPR 1.28, 95% CI 1.07-1.53, p=0.007) and lower education (aPR 1.64, 95% CI 1.33-2.01, p<0.001) remained independent determinants. Hypertension (aPR 1.21, 95% CI 1.02-1.43, p=0.03) and diabetes mellitus (aPR 1.27, 95% CI 1.07-1.52, p=0.008) were also significant. A history of stroke or TIA was one of the strongest independent determinants (aPR 1.89, 95% CI 1.52-2.35, p<0.001). Depression retained a significant association (aPR 1.48, 95% CI 1.16-1.90, p=0.002). Finally, an ACB score ≥3 was independently linked with higher dementia prevalence (aPR 1.36, 95% CI 1.09-1.70, p=0.005).

**Table 3:** Crude and adjusted PR of factors associated with dementia Crude PRs were obtained from univariable analysis, while adjusted PRs were derived from multivariable Poisson regression models including all listed covariates. PR: prevalence ratio; TIA: transient ischemic attack; ACB: Anticholinergic Cognitive Burden

Factor	Crude PR	95% CI	p-value	Adjusted PR	95% CI	p-value
Age ≥80 years	2.98	2.45–3.63	<0.001	2.41	1.92–3.01	<0.001
Female sex	1.24	1.05–1.47	0.01	1.28	1.07–1.53	0.007
Education ≤6 years	1.89	1.56–2.29	<0.001	1.64	1.33–2.01	<0.001
Hypertension	1.35	1.14–1.59	<0.001	1.21	1.02–1.43	0.03
Diabetes mellitus	1.41	1.19–1.67	<0.001	1.27	1.07–1.52	0.008
Stroke/TIA history	2.41	1.99–2.92	<0.001	1.89	1.52–2.35	<0.001
Depression	2.12	1.71–2.62	<0.001	1.48	1.16–1.90	0.002
ACB score ≥3	1.82	1.45–2.29	<0.001	1.36	1.09–1.70	0.005

Sensitivity analyses using alternative case definitions were conducted to test the robustness of the prevalence estimates. Defining dementia more strictly, either as two separate ICD-10 codes or as a combination of a diagnostic code with an anti-dementia prescription, yielded a prevalence of 12.0% (95% CI 11.0-13.1), which was very similar to the main analysis (12.5%). When restricted only to patients who had both a diagnostic code and a prescription, the prevalence was 11.8% (95% CI 10.8-12.9). The associations of age, sex, education, comorbidities, and ACB score with dementia remained significant and consistent with the primary model. These results indicate that our findings are robust (Table 4).

**Table 4:** Sensitivity Analysis of Dementia Prevalence ICD: International Classification of Diseases; CI: confidence interval

Case definition for dementia	Prevalence % (95% CI)
Primary definition (ICD code, prescription, or test)	12.5 (11.5–13.6)
≥2 ICD-10 codes on separate visits	12.0 (11.0–13.1)
ICD-10 code + prescription for anti-dementia drug	11.8 (10.8–12.9)

## Discussion

In this clinic-based study of older adults attending outpatient services in a tertiary care hospital, we found that the prevalence of dementia was 12.5%, with the highest burden observed in neurology and geriatric clinics. This estimate is higher than community-based studies in Thailand, which reported prevalence ranging from 2.8% to 8.1% [4,5], but consistent with the expectation that tertiary care populations represent older and more comorbid patients. Our findings align with reports from other Asian countries where hospital-based prevalence rates are typically greater than those from community surveys [3]. In our study, most dementia cases were due to Alzheimer’s disease, followed by vascular dementia. This pattern underscores the combined impact of neurodegenerative and vascular causes among older adults in Thailand. Notably, the proportion of vascular dementia appeared higher than that reported in many Western cohorts, which is in line with the greater prevalence of vascular risk factors observed in Asian populations [3,19-21].

The observed prevalence is comparable to global estimates in hospital populations (10% to 20%) and underscores the importance of routine screening in outpatient clinics [1,2]. The clinic-specific variation, with the neurology clinic showing the highest prevalence, likely reflects referral bias, as patients with cognitive or neurological complaints are preferentially directed there. In contrast, the lower prevalence in internal medicine clinics may indicate under-recognition of dementia in general medical practice.

Consistent with prior literature, advanced age was the strongest risk factor for dementia [6,7]. Female sex was also independently associated, a finding that has been attributed both to women’s longer life expectancy and possible sex-related biological vulnerability [9]. Low education emerged as another strong predictor, supporting the cognitive reserve hypothesis that higher education may buffer against clinical expression of dementia [6].

Vascular comorbidities, hypertension, diabetes, and stroke were robustly associated with dementia in our analysis. These findings reinforce the concept of “vascular contributions to cognitive impairment and dementia” (VCID), emphasizing the need for optimal management of vascular risk factors to reduce dementia burden [7,8]. Depression was also strongly linked, consistent with evidence that late-life depression may act both as a risk factor and an early manifestation of dementia [9].

Importantly, we identified polypharmacy and high anticholinergic burden (ACB score ≥3) as independent correlates of dementia. This highlights the role of potentially inappropriate medications in exacerbating cognitive decline, echoing previous studies linking anticholinergic exposure to incident dementia [16-18]. These findings suggest that medication review should be a routine component of dementia risk reduction in tertiary care.

Our findings have several implications. Our findings point toward the need for more systematic dementia screening in tertiary outpatient settings. Studies have repeatedly shown that cognitive decline is markedly under-recognized in general internal medicine practice. One study in a Brazilian tertiary hospital found that only 16.3% of patients later diagnosed with dementia had any cognitive complaints documented [22,23]. Similarly, international evidence suggests consistently low detection rates in primary care environments [24]. Second, integrated management of vascular risk factors should be prioritized as part of dementia prevention strategies [16-18]. Third, deprescribing anticholinergic medications where appropriate may represent a modifiable target for reducing cognitive decline. Finally, the study underscores the importance of training general internists and other clinicians to recognize dementia, given the lower prevalence identified in internal medicine clinics, which may reflect underdiagnosis [25-27].

This study has several strengths, including its large sample size, the ability to draw on different types of information within the hospital database (ICD codes, prescription records, and cognitive test results), and the inclusion of data from multiple outpatient clinics. However, several limitations should be acknowledged. First, as a retrospective cross-sectional design, causal inference cannot be established. Second, our case ascertainment relied in part on administrative codes and prescription records, which may have resulted in some misclassification of dementia cases. Sensitivity analyses, however, indicated that the overall estimates were robust. It is also possible that dementia was under-recognized in the internal medicine clinic, since not all patients without a diagnostic code or dementia medication underwent cognitive testing at the index visit. Third, there may still be residual confounding from lifestyle factors that were not captured in our dataset, such as smoking, alcohol use, and physical activity. We also reviewed vitamin B12, folate, and TSH results to help rule out reversible causes of dementia, but these tests were missing for many patients and therefore could not be included in the analysis of determinants. Finally, the study was conducted at a single tertiary care hospital, and findings may not be generalizable to community or primary care settings.

## Conclusions

This study demonstrated that dementia is highly prevalent among older adults attending outpatient clinics in a tertiary care hospital in Thailand, with rates exceeding those reported in community-based studies. Advanced age, female sex, and low education were strong non-modifiable correlates, while vascular comorbidities, depression, and high anticholinergic burden emerged as significant modifiable factors associated with dementia. These findings underscore the importance of integrating routine dementia screening into outpatient services, prioritizing aggressive management of vascular risk factors, and implementing regular medication reviews to minimize anticholinergic exposure. Targeted strategies addressing these risk factors may help reduce the burden of dementia in high-risk hospital populations and inform resource allocation in similar clinical settings.
